# Mixed Edge Activators in Ibuprofen-Loaded Transfersomes: An Innovative Optimization Strategy Using Box–Behnken Factorial Design

**DOI:** 10.3390/pharmaceutics15041209

**Published:** 2023-04-11

**Authors:** João Vieira, Jéssica Castelo, Marta Martins, Nuno Saraiva, Catarina Rosado, Catarina Pereira-Leite

**Affiliations:** 1 CBIOS—Universidade Lusófona’s Research Center for Biosciences & Health Technologies, Campo Grande 376, 1749-024 Lisboa, Portugal; joao.manuel.vieira@ulusofona.pt (J.V.); marta.filipa.martins@ulusofona.pt (M.M.); nuno.saraiva@ulusofona.pt (N.S.); catarina.rosado@ulusofona.pt (C.R.); 2 Department of Biomedical Sciences, University of Alcalá, Ctra. Madrid-Barcelona Km. 33.600, Alcalá de Henares, 28871 Madrid, Spain; 3 School of Health Sciences and Technologies, Universidade Lusófona, Campo Grande 376, 1749-024 Lisboa, Portugal; 4 LAQV, REQUIMTE, Departamento de Ciências Químicas, Faculdade de Farmácia, Universidade do Porto, Rua de Jorge Viterbo Ferreira 228, 4050-313 Porto, Portugal

**Keywords:** transfersomes, nanovesicular systems, ibuprofen, nonionic surfactants, Box–Behnken factorial design, skin delivery

## Abstract

Transfersomes have been highlighted as an interesting nanotechnology-based approach to facilitate the skin delivery of bioactive compounds. Nevertheless, the properties of these nanosystems still need to be improved to enable knowledge transfer to the pharmaceutical industry and the development of more efficacious topical medicines. Quality-by-design strategies, such as Box–Behnken factorial design (BBD), are in line with the current need to use sustainable processes to develop new formulations. Thus, this work aimed at optimizing the physicochemical properties of transfersomes for cutaneous applications, by applying a BBD strategy to incorporate mixed edge activators with opposing hydrophilic–lipophilic balance (HLB). Tween^®^ 80 and Span^®^ 80 were used as edge activators and ibuprofen sodium salt (IBU) was selected as the model drug. After the initial screening of the IBU solubility in aqueous media, a BBD protocol was implemented, and the optimized formulation displayed appropriate physicochemical properties for skin delivery. By comparing the optimized transfersomes to equivalent liposomes, the incorporation of mixed edge activators was found to be beneficial to upgrade the storage stability of the nanosystems. Furthermore, their cytocompatibility was shown by cell viability studies using 3D HaCaT cultures. Altogether, the data herein bode well for future advances in the use of mixed edge activators in transfersomes for the management of skin conditions.

## 1. Introduction

Despite the accessibility of the skin, the largest and most superficial organ of the human body, the treatment of many cutaneous disorders still remains a challenge. For instance, in the case of inflammatory skin diseases, such as atopic dermatitis and psoriasis, no curative options are available, and the topical treatment is based on reducing the disease severity using drugs (e.g., glucocorticoids and calcineurin inhibitors) with poor safety profiles [1,2]. From a different perspective, rare skin conditions related to DNA disorders, namely, xeroderma pigmentosum (XP), Cockayne syndrome, and trichothiodystrophy, require a demanding daily care scheme to reduce the exposure to UV light and the occurrence of xerosis [3]. Considering XP, daily care formulations mainly aim at reducing the incidence of skin cancer, thus the topical application of anti-cancer drugs seems to be a promising approach to control the cell malignant transformation [3,4]. However, once again, safety issues may underlie this preventive measure.

In this context, the search for new drug delivery systems (DDSs) to improve the efficacy and safety of skin therapies has been increasing in recent years [5,6]. Topical formulations have become increasingly complex to meet the requirements of drug delivery to epidermal, dermal, and appendageal target sites, or even to the underlying tissue [7]. Despite representing an interesting option, this route of administration has an important drawback related to the skin barrier, in particular that opposed by the stratum corneum, which significantly limits drug penetration. Altogether, the therapeutic agents and the delivery systems used to administer them must fit in a narrow window of physicochemical properties to overcome the skin hurdles and reach target sites [8,9]. Nanotechnology-based DDSs have been highlighted as useful tools in skin delivery, as they can be designed according to the physicochemical properties of the drug and can also modulate the skin barrier function to enhance drug permeation [10]. Phospholipids combined with ethanol or water can be used in carriers for drugs with different profiles, surpassing bioavailability, solubility, or toxicity constraints [11,12]. Within nanotechnology-based DDSs, conventional lipidic vesicles (liposomes) were the first systems to be suggested for skin delivery [13]. However, the reported limitations of liposomes, such as the poor storage stability and limited skin penetration [14], triggered the development of a new class of nanovesicular systems called transfersomes. These emerged as an innovative way to enhance vesicle properties, as they are ultra-elastic vesicles composed of phospholipids and edge activators (EAs), which enable deformation of the lipid bilayer and a reduction in vesicle size without compromising drug loading [15]. As reported by Cevc and Blume, transfersomes cross intact skin owing to hydration force and the transdermal osmotic gradients [16]. Regio-selective delivery can be achieved by these carriers, which have been reported to become almost exclusively located in the viable skin region [17].

The EAs used to produce transfersomes have a great impact on the physicochemical properties of these vesicles. Substantial evidence shows their ability to enhance drug solubility and entrapment efficiency, as well as to modulate the elasticity, permeability, and stability of the vesicles [18]. As a result, the type of EA and the EA/lipid weight ratio are considered crucial for the features and performance of the transfersomes [19]. Nonionic surfactants have been widely used as EAs in transfersomes and their hydrophilic–lipophilic balance (HLB) is one of their most impactful characteristics [18,20,21]. Moreover, the combination of nonionic surfactants with opposing HLB values was recently suggested as a valuable strategy to further modulate the properties of transfersomes [22] and as a step forward to optimize these nanovesicular systems.

Having in mind the importance of using sustainable processes for the development of new formulations, a quality-by-design (QbD) approach was selected to be implemented in this study. Using this strategy, the final formulation can be optimized with improved characteristics for skin delivery, while spending fewer resources and limiting the costs. Among the response surface methodologies, Box–Behnken factorial design (BBD) stood out as it is considered to be highly efficient and economical to perform a multivariate analysis [23,24] and it has been widely used to optimize nanosystems [25,26,27].

The main objective of this work was, therefore, the optimization of transfersomes made of mixed EAs with opposing HLB values employing a BBD strategy. The EAs selected for this study were Tween^®^ 80 and Span^®^ 80, as they are widely used to produce transfersomes and display disparate HLB values (15 and 4.3, respectively). Ibuprofen sodium salt (IBU) was selected as a hydrophilic model drug to be encapsulated in the developed transfersomes and the drug solubility in aqueous media was firstly evaluated in a preformulation study. After defining the optimized formulation using BBD, a comparative study between transfersomes and the equivalent liposomes was performed in terms of their physicochemical properties, stability under refrigerated conditions, and impact of the vesicles on the viability of HaCaT spheroids. Overall, this work showed that the incorporation of mixed EA with opposing HLB values in transfersomes is a valuable approach, as these innovative vesicular nanosystems displayed suitable properties for skin delivery with improved colloidal stability over time.

## 2. Materials and Methods

### 2.1. Materials

Transfersomes and liposomes were prepared using soy phosphatidylcholine acquired from Alfa Aesar (Kandel, Germany), Span^®^ 80 from Merck (Darmstadt, Germany), chloroform from Scharlab S.L. (Sentmenat, Spain), methanol from Carlo Erba Reagenti SpA (Rodano, Italy), and Tween^®^ 80 and IBU from Sigma-Aldrich (Saint Louis, MO, USA). Phosphate-buffered saline (PBS, pH 7.4) was prepared as previously reported [23].

For the cell viability studies, Dulbecco’s Modified Eagle’s Medium Low Glucose (DMEM), Fetal Bovine Serum (FBS), and phosphate-buffered saline without calcium and magnesium X1 (PBS) were obtained from VWR International and were provided by Biowest (Nuaillé, France). The Trypsin-EDTA (0.05%) phenol red was received from Gibco (Grand Island, NY, USA), Penicillin-Streptomycin antibiotic (PEN-STREP) from Corning (Glendale, AZ, USA), and Propidium iodide (PI) from Molecular Probes (Eugene, OR, USA).

### 2.2. Solubility Studies

Solubility studies of IBU were performed as previously reported with some modifications [28]. Saturated solutions of IBU, in the presence of excess of solute, were prepared in bidistilled water or PBS buffer, in triplicate, and then stirred for 72 h at 25 ± 2 °C using a horizontal orbital shaker (IKA VIBRAX VXR^®^, LTF Labortechnik GmbH & Co., Bodensee, Germany). Later, all solutions were filtered, and the IBU solubility was quantified through a calibration curve method based on the UV/Vis data, at the maximum absorption wavelength of the drug in the studied solvents (264 nm) [29], acquired using the Evolution^®^ 300 UV/Visible spectrophotometer (Thermo Scientific, Hertfordshire, UK).

### 2.3. Preparation of Transfersomes and Liposomes

The thin-film hydration method followed by sonication was used to prepare transfersomes and liposomes, as previously described [22,28]. Initially, the lipid films were prepared by dissolving soy phosphatidylcholine in a mixture of chloroform/methanol (3:1, *v*/*v*), in the absence (liposomes) or presence of two EAs, Tween^®^ 80 and Span^®^ 80 (transfersomes). The organic solvents were then removed first using a rotary evaporator (40 °C, 90 rpm, 20 min) and then by leaving the lipid films under a vacuum atmosphere for 2 h. After this, vesicles were produced by adding an IBU buffered solution or PBS buffer under vortexing, with a subsequent sonication (50% amplitude, 10 min) using a Q125 Sonicator (QSonica Sonicators, Newtown, CT, USA). Finally, the formulations were left in a horizontal shaker (200 rpm) to equilibrate for 30 min.

### 2.4. Optimization of IBU-Loaded Transfersomes

A Box–Behnken factorial design (BBD), considering 15 runs, 3 factors, and 3 levels, was employed to optimize the IBU-loaded transfersomes. In this quality-by-design (QbD) strategy, the defined factors were the lipid concentration (X_1_), the Tween^®^ 80/Span^®^ 80 ratio (X_2_), and the concentration of IBU (X_3_). Along with this, the evaluated responses were the vesicle size (Vs or Y_1_), the polydispersity index (PDI or Y_2_), the encapsulation efficiency (EE or Y_3_), and the loading capacity (LC or Y_4_). Based on our preliminary results and literature data [28,30], three different levels per factor were defined to be tested, as well as the desirable criteria to be considered for each response (Table 1).

The BBD results were run in STATISTICA^®^ software (Statsoft, Tulsa, OK, USA) to estimate the optimum level for each factor to achieve the higher desirability value and, consequently, to provide the optimized formulation. Finally, to validate this QbD approach, three replicates of the optimized formulation were produced and then physicochemically characterized to compare the experimental responses to the theoretical data anticipated by the BBD.

### 2.5. Characterization of Transfersomes and Liposomes

#### 2.5.1. Vesicle Size, Polydispersity Index, and Zeta Potential

After the production step, the formulations were diluted 1:20 (*v*/*v*) with distilled water and then characterized in terms of vesicle size (Vs) and polydispersity index (PDI) by dynamic light scattering (DLS), using a Delsa^TM^ Nano C equipment (Beckman Coulter, Brea, CA, USA), and zeta potential (ZP) by phase analysis light scattering (PALS) using the NanoBrook Omni equipment (Brookhaven Instruments, Holtsville, NY, USA). For DLS and PALS data acquisition, 1 run with 70 cycles and 1 run with 30 cycles were performed, respectively. All measurements were carried out in triplicate for each sample at 23 ± 2 °C.

#### 2.5.2. Encapsulation Efficiency and Loading Capacity

The encapsulation efficiency (EE) and loading capacity (LC) of the vesicles were estimated by an indirect assay based on the quantification of the free drug in the external medium that surrounds the vesicles. Each sample was diluted 1:10 (*v*/*v*) with PBS buffer and then transferred to a VIVASPIN^®^ 500 centrifuge tube (10 KDa, Sartorius, Goettingen, Germany) with a subsequent centrifugation at 14,000× *g* for 40 min in a Hermle Z323 K centrifuge (Hermle LaborTechnik, Wehingen, Germany). Afterwards, the supernatant, corresponding to the non-loaded IBU fraction, was diluted in PBS buffer (1.5:8.5) and the absorbance was determined at 222 nm using an Evolution^®^ 300 UV/Visible spectrophotometer (Thermo Scientific, Hertfordshire, UK). After calculating the concentration of IBU in the non-loaded fraction ([IBU_NL_]) based on a previously determined calibration curve, it was possible to calculate the EE and LC, knowing the total concentration of IBU added to prepare the vesicles ([IBU_T_]), as well as the total concentration of soy phosphatidylcholine used to prepare the vesicles ([PC_T_]), according to the equations below:

(1) $$\%\;\mathrm{EE}=\;\frac{\left[{\mathrm{IBU}}_{T}\right]-\left[{\mathrm{IBU}}_{\mathrm{NL}}\right]}{\left[{\mathrm{Ibu}}_{T}\right]}\;\times \;100$$

(2) $$\%\;\mathrm{LC}=\frac{\left[{\mathrm{IBU}}_{T}\right]-\left[{\mathrm{IBU}}_{\mathrm{NL}}\right]}{\left[{\mathrm{PC}}_{T}\right]}\times 100$$

### 2.6. Stability Studies

Four distinct formulations were tested in terms of physicochemical stability, namely IBU-loaded and unloaded transfersomes and IBU-loaded and unloaded liposomes. After preparation, all formulations were stored at 5 ± 3 °C for two months in line with ICH guidelines and were characterized at different days (7th, 15th, 30th, and 60th) after production in terms of Vs, PDI, ZP, EE, and LC, as previously described (Section 2.5). The choice of performing stability studies in refrigerated conditions was based on the well-known nature of phospholipids to undergo chemical and physical instability at room temperature [31,32].

### 2.7. Three-Dimensional Cell Culture

Human immortalized keratinocytes (HaCaT cells, CLS Cell Lines Service GmbH, Eppelheim, Germany) were maintained in complete media (DMEM with 10% FBS and Pen/Strep). HaCaT 3D cultures were generated in 96-well plates coated with 50 µL of 1% agar (Alfa Aesar, Kandel, Germany) [33]. Briefly, 1.5 × 10^4^ cells were added to each well in complete culture media and cultures were incubated for 48 h to obtain cell spheroids (one per well).

### 2.8. Viability Studies

Propidium iodide (10 µg/mL) was added to the 3D cell cultures previously incubated with vesicles or controls for 24 h, and incubated for 20 min at 37  °C, under a 5% CO_2_ humidified atmosphere [34]. As dead cells are permeable to PI, an increase in PI fluorescence is observed in dead cells. Image acquisition was performed on a Zeiss Axio Observer microscope (White Plains, NY, USA) with a ×20 objective using ZEN software. The average PI fluorescence intensity of the spheroid area was measured with ZEN software. Phase contrast was used to define the spheroid area. A minimum of four spheroids were used per condition in each assay and three independent assays were performed.

### 2.9. Statistical Analysis

The statistical analysis of the data from BBD was performed with STATISTICA^®^ software (Statsoft, Tulsa, OK, USA), while all other data were analyzed using GraphPad Prism (GraphPad Software Inc., San Diego, CA, USA). After running normality and homogeneity tests, statistical differences between the groups to be compared were calculated by one-way ANOVA followed by Turkey’s multiple comparisons (transfersomes versus liposomes characterization) or two-way ANOVA followed by Bonferroni post hoc test (stability studies) or Tukey’s multiple comparison test (cell viability studies). The statistically significant results were considered for *p*-values < 0.05.

## 3. Results

### 3.1. Solubility of Ibuprofen Sodium Salt in Aqueous Media

To screen the most appropriate aqueous media to prepare the lipid nanovesicles, the solubility of ibuprofen sodium salt (IBU) was determined in bidistilled water (pH 5.5) and PBS buffer (pH 7.4). The IBU solubility was superior in bidistilled water (285 ± 10 mg/mL) compared with in PBS buffer (161 ± 12 mg/mL). As IBU is a weak acid (pKa = 4.85, according to pKa Plugin MarvinSketch 20.9.0, ChemAxon), IBU solubility in PBS was expected to be higher than in water, as the number of drug molecules ionized at pH 7.4 is greater than at pH 5.5. However, the obtained result in PBS can be attributed to a salting out phenomenon, by which a reduction in the IBU solubility may occur as a result of the presence of a substantial concentration of salts in the buffer solution.

Despite the greater solubility of IBU in bidistilled water, PBS buffer was the chosen solvent to prepare the lipid nanovesicles. The solubility in both aqueous media is considerably high, thus enabling the drug to remain in the aqueous compartment of the nanovesicles. Additionally, the use of PBS buffer is always advantageous not only because it more closely mimics the body internal environment, but also because its salt composition may contribute to the colloidal stability of the nanovesicles.

### 3.2. Optimization of IBU-Loaded Transfersomes by a Box–Behnken Factorial Design

As explained previously, the QbD approach BBD was employed to develop and optimize IBU-loaded transfersomes. For this purpose, fifteen formulations were produced to test three levels of each selected factor: the lipid concentration (X_1_), the Tween^®^ 80/Span^®^ 80 ratio (X_2_), and the concentration of IBU (X_3_), as displayed in Table 2. After preparation, all formulations were characterized in terms of vesicle size (Vs or Y_1_), polydispersity index (PDI or Y_2_), encapsulation efficiency (EE or Y_3_), and loading capacity (LC or Y_4_), and the obtained results are presented in Table 2. It is noteworthy that the obtained responses were within the previously selected desirability criteria (Table 1): 85 nm < Vs < 172 nm; 0.17 < PDI < 0.29; 10% < EE < 38%; and 0.08% < LC < 0.88%.

To analyze the obtained data, the two-way interaction (linear × quadratic) was chosen as the fitting model, according to the R^2^ values obtained for each response (Table 3), confirming the relevance of the cubic regression model as an optimizing strategy. The influence of each factor and their interactions (linear or quadratic) on each response is depicted in Table 3. It is important to note that a positive or negative interaction coefficient means that a certain factor or their interactions exert a synergistic or antagonistic effect (respectively) on the evaluated response. Considering *p <* 0.05 as statistical significance, the results demonstrate that the Tween^®^ 80/Span^®^ 80 ratio (X_2_) had a negative effect on Vs, i.e., the higher the amount of Tween^®^ 80, the smaller the vesicle size. Additionally, synergistic effects were observed for the linear and quadratic interactions of the lipid concentration and the surfactants ratio (X_1_X_2_ and X_1_^2^X_2_, respectively), as well as for the quadratic interaction between the lipid concentration and IBU concentration (X_1_^2^X_3_). Regarding PDI, negative effects were observed for several quadratic interactions of factors (X_2_^2^, X_3_^2^, and X_1_X_2_^2^), as well as for a linear one (X_1_X_2_). However, a positive effect for the Tween^®^ 80/Span^®^ 80 ratio (X_2_) as well as for the IBU concentration (X_3_) was observed. Consequently, increasing the levels of these factors leads to higher PDI values. Concerning EE, only the surfactants ratio was found to cause a linear or quadratic antagonistic effect (X_2_ and X_2_^2^, respectively), thus, the higher the amount of Tween^®^ 80, the smaller the EE. Finally, factors like the lipid concentration (linear or quadratic, X_1_ or X_1_^2^) and the Tween^®^ 80/Span^®^ 80 ratio (linear or quadratic, X_2_ or X_2_^2^) displayed negative effects on LC, while the IBU concentration (X_3_) had a positive impact.

**Figure 1:**
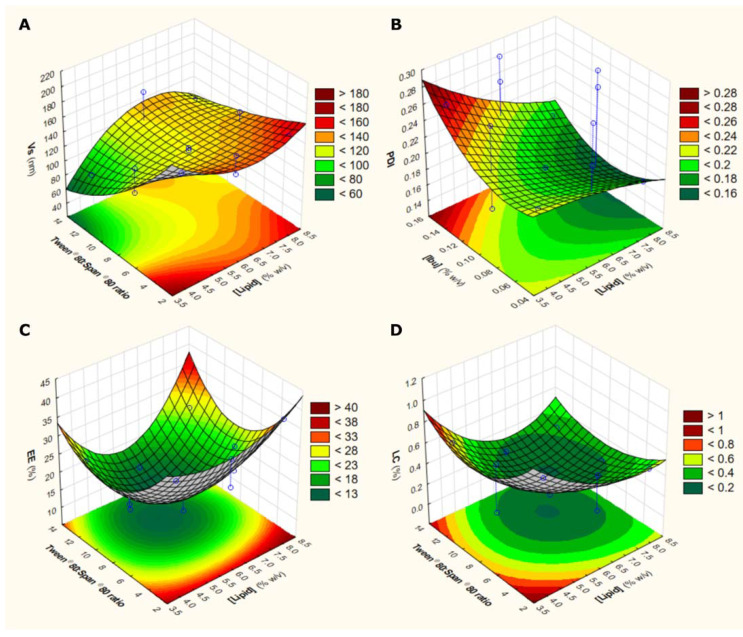
Response surface plots in 3D for Vs (**A**), PDI (**B**), EE (**C**), and LC (**D**). The effects of factors on each response are highlighted with various response degree levels, from dark green (lowest level) to dark red (highest level).

Three-dimensional response surface plots (Figure 1) are presented to illustrate the statistically relevant effects of two intervening factors on a specific response, keeping the third factor constant at the middle level (0). Considering Vs (Figure 1A), it is clear that higher values are obtained when lower Tween^®^ 80/Span^®^ 80 ratios are combined with high lipid concentrations. Moreover, by decreasing the IBU concentration, it is possible to obtain lower values of PDI, illustrating the positive effect of the X_3_ factor (Figure 1B). Considering EE and LC (Figure 1C and 1D, respectively), a negative effect of X_2_ can be observed_,_ as the highest values of EE are obtained for the lowest values of the Tween^®^ 80/Span^®^ 80 ratio. In the case of LC (Figure 1D), the antagonistic effect of the lipid concentration is also visible, as the lower the X_1_ value, the higher the LC.

Based on the significant effects of factors, and of their relationships, on the observed responses, the STATISTICA^®^ software predicted the optimum values for each factor to prepare transfersomes with the pre-defined desirable characteristics (Table 1), as detailed in Table 4. Moreover, the theoretical values to be obtained for each response were also predicted (Table 4). To validate the implemented BBD, three independent formulations were produced, and the obtained responses were compared to the theoretical data (Table 4). The optimized transfersomes displayed interesting properties considering the predefined desirable criteria, with Vs of 166 nm, PDI of 0.23, EE of 34%, and LC of 1.1%. These values are in reasonable accordance with the theoretical predicted responses; thus, BBD was found to be a trustworthy and useful tool to optimize transfersomes made of mixed EAs to load IBU.

### 3.3. Transfersomes versus Liposomes: Characterization and Storage Stability

To study the effect of IBU loading and/or surfactants’ composition on the physicochemical properties of the nanovesicular systems, loaded and unloaded transfersomes (Unl-TR and IBU-TR, respectively) and the equivalent liposomes (Unl-Lip and IBU-Lip, respectively) were produced in triplicate and characterized. As can be seen in Table 5, transfersomes and liposomes displayed similar physicochemical properties and no significant differences were found between the properties of IBU-TR and IBU-Lip. In contrast, unloaded liposomes were smaller than both IBU-loaded and unloaded transfersomes. These results show that the incorporation of IBU and/or surfactants increases the vesicular size, while no other property is significantly affected.

**Figure 2:**
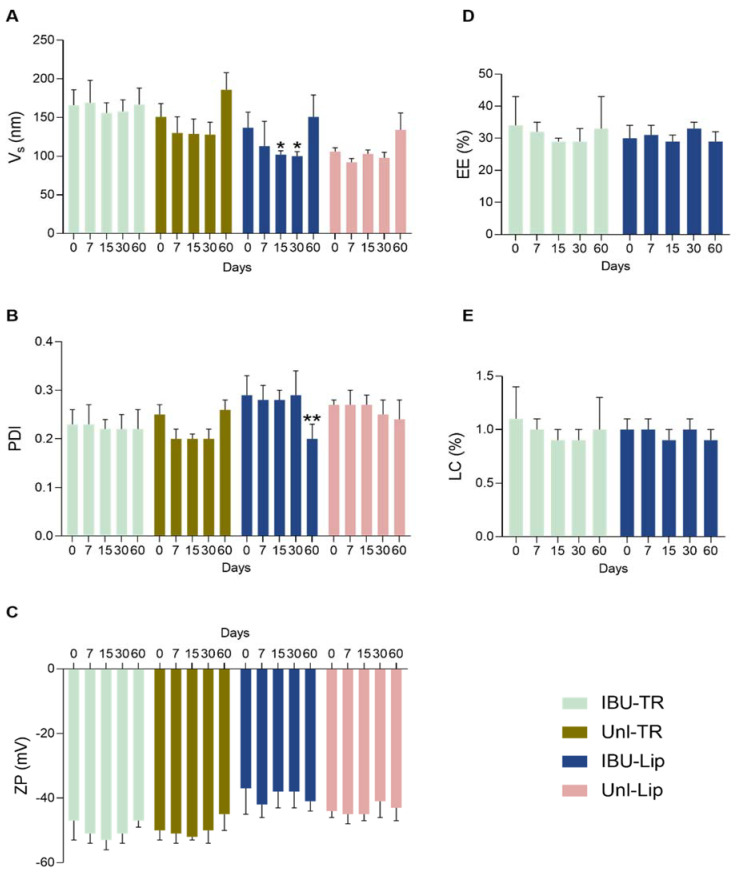
Storage stability under refrigerated conditions (5 ± 3 °C) up to 60 days of unloaded and IBU-loaded transfersomes (Unl-TR and IBU-TR, respectively) and liposomes (Unl-Lip and IBU-Lip, respectively) in terms of the following: (**A**) vesicle size (Vs); (**B**) polydispersity index (PDI); (**C**) zeta potential (ZP); (**D**) encapsulation efficiency (EE); and (**E**) loading capacity (LC). * *p* < 0.05 and ** *p* < 0.01 compared with synthesis day—T0 (two-way ANOVA, Bonferroni’s multiple comparison test).

Afterwards, the storage stability under refrigerated conditions of all four formulations was assessed for 60 days and characterized in terms of Vs, PDI, zeta potential (ZP), EE, and LC after 7, 15, 30, and 60 days (Figure 2). When comparing the values between production day (T0) and the remaining days of storage, no significant differences were observed for ZP, EE, and LC values (Figure 2C–E) for both IBU-loaded and unloaded transfersomes or liposomes. In contrast, IBU-loaded liposomes displayed a significant decrease in Vs after 15 days of storage and in PDI after 60 days of storage (Figure 2A,B). These results suggest that the presence of surfactants on transfersomes stabilizes the size and PDI of vesicles over time. Thus, the developed transfersomes made of mixed EAs have improved physicochemical properties in comparison with the equivalent liposomes and are stable for 60 days under refrigerated conditions.

### 3.4. Transfersomes versus Liposomes: Viability Studies with HaCaT Spheroids

**Figure 3:**
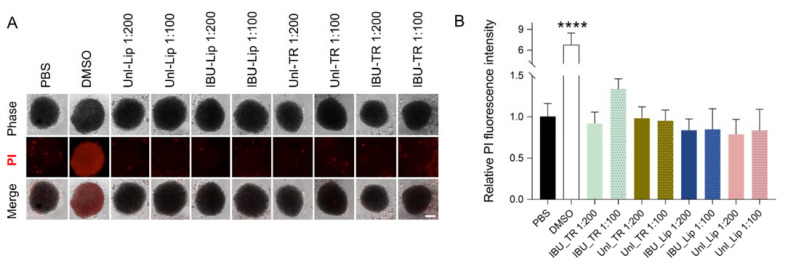
Unloaded and IBU-loaded transfersomes (Unl-TR and IBU-TR, respectively) and liposomes (Unl-Lip and IBU-Lip, respectively) did not induce cell death of 3D HaCaT cell cultures. (**A**) Representative images of 3D HaCaT cultures incubated for 24 h with the indicated transfersomes and liposomes, vehicle (PBS), or a cell death inducer (10% DMSO). (**B**) The summarized results of cell viability studies are shown as mean relative fluorescence intensity ± SD from three independent experiments. **** *p* < 0.0001 compared with vehicle-treated cells (two-way ANOVA, Tukey’s multiple comparison test). Scale bar 100 µm.

To assess the impact of the nanovesicular systems produced in this study on the cell viability of human keratinocytes (HaCaT), a 3D cell spheroid model was used. Considering the differences in the permeability of transfersomes and liposomes, the use of a 3D culture method (cell spheroid) represents a more relevant and physiological condition in which the impact of these particles on cell viability can be assessed. After an incubation of the spheroids with 1:100 and 1:200 dilutions of IBU-loaded and unloaded transfersomes and liposomes for 24 h, no significant increase in the levels of PI fluorescence was measured (Figure 3), thus indicating that the formulations did not increase the number of dead cells in the spheroid.. These data indicate that, in the conditions used, none of the formulations under study have a significant impact on the induction of cell death.

## 4. Discussion

This work was focused on using a QbD strategy to optimize transfersomes loaded with IBU composed of mixed EAs with contrasting HLB values—Tween^®^ 80 and Span^®^ 80. A preformulation study on IBU solubility was performed to select a suitable aqueous solvent for the production of the nanosystem. The solubility in PBS was found to be suitable for the envisioned drug loading, and this was the chosen media. These data highlight the importance of solubility studies in the preformulation phase, as key biopharmaceutical characteristics such as drug solubility and permeability are considerably sensitive to the experimental medium composition [35].

The use of optimization strategies such as BBD has proved to be beneficial to obtain DDSs with improved features, at the same time lowering costs, resources, and time consumption [36]. BBD had already been employed to optimize transfersomes for the delivery of various compounds such as apigenin, rutin, and gallic acid [26,28,30], but to the best of our knowledge, this was the first time this strategy was used to optimize transfersomes made of mixed EAs. Additionally, in this study, a BBD strategy was developed to produce transfersomes with suitable physicochemical properties (Vs, PDI, ZP, EE, and LC) for the skin delivery of a model drug—IBU. The results show that the BBD was successfully employed, as the obtained transfersomes displayed physicochemical properties in line with the desirability criteria. The desirability criteria considered in this study (Table 1) were based on the following points: (a) smaller vesicular nanosystems display enhanced skin permeation [37,38]; (b) vesicular nanosystems should display uniform sizes with PDI values lower than 0.3 [37]; and (c) the higher the EE and LC, the lower the production costs. The optimized transfersomes were then composed of a 2.5:12.5:85 mixture of Tween^®^ 80/Span^®^ 80/phosphatidylcholine and showed a Vs of 166 nm, a PDI of 0.23, an EE of 34%, and a LC of 1.1%. This result is a clear breakthrough considering the previously reported paper on the development of ibuprofen-loaded transfersomes [39], particularly in terms of obtaining vesicles in the nanoscale under 300 nm to ensure skin penetration [37]. This improvement may result not only from the higher EA/phosphatidylcholine ratio, but also from the synergy resulting from combining two surfactants with different HLB values. Indeed, it was already reported that an adequate surfactant ratio is essential for the production of transfersomes [19,23] and that the HLB of the surfactant has a significant effect on the size of the transfersomes, mainly owing to their affinity toward phospholipids [40]. On the other hand, the HLB of the surfactant also affects the drug incorporation into the transfersomes, and surfactants with low HLB values usually improve the encapsulation of lipophilic drugs [18]. This fact justifies the modest EE obtained in this study (34%), as the optimized transfersomes had a higher ratio of Span^®^ 80 (HLB of 4.3) and the sodium salt form of ibuprofen used in this study has a considerable affinity to water. Indeed, the EE obtained herein was relevant because it is often challenging to load hydrophilic compounds into vesicular systems with the thin film hydration method [41]. Overall, it should be stressed that the use of mixed nonionic surfactants to produce transfersomes was a valuable strategy to obtain vesicles with optimum size and size uniformity for skin delivery, together with reasonably good drug loading.

To analyze the impact of adding the EAs and IBU to the vesicles, loaded and unloaded transfersomes and the more conventional liposomes (without EA) were prepared and characterized. The obtained results show that the incorporation of IBU and/or EA increases the size of the nanovesicles, with no effects on the other evaluated properties, but both transfersomes and liposomes exhibited suitable nanosizes. This result was expected because Span^®^ 80, the predominant EA in the mixture, is described to have a high affinity for the phospholipid bilayer, thereby causing an increase in the size of the vesicles [18,22]. On the other hand, IBU was already described to be capable of interacting with the headgroup regions of phosphatidylcholine bilayers [42], which may also contribute to the increment in the vesicle diameter. Moreover, all formulations were submitted to a storage stability test of up to 2 months, which revealed that the presence of EA provided an improved colloidal stability, and thus a superior performance of transfersomes, particularly in terms of vesicle size and PDI. It is noteworthy that the optimization of the EA mixture to produce transfersomes enabled a better stability profile than that previously reported for transfersomes made of Tween^®^ 80, Span^®^ 80, or the Tween^®^ 80/Span^®^ 80 50:50 mixture [22]. This outcome suggests that the optimization of the EA ratio has a crucial role in modulating the transfersomes physicochemical properties over time.

Besides this, to explore the impact of the nanovesicular systems on the viability of human keratinocytes, a 3D cell spheroid model based on the HaCaT cell line was applied. Three-dimensional models of HaCaT cells can be used as simplified models of normal or abnormal epidermis [43,44]. The use of 3D cell cultures better mimics the physiological environment found by nanovesicular systems in the in vivo pathway when compared with the traditional 2D systems. This is particularly relevant in the case of transfersomes, given the improved ability of these particles to penetrate intercellular spaces. Despite this fact, to the best of our knowledge, 3D HaCaT spheroids are not typically used to evaluate the impact of lipid-based nanoparticles on cell viability. Although this model presents clear improvements, most studies involving transfersomes still use the widespread 2D HaCaT culture method for cell viability assessment [45,46]. The results obtained here evidenced that loaded and unloaded transfersomes and liposomes did not impact cell death. This is similar to what has been described for other transfersomes in HaCaT 2D cultures [45,46]. This first assessment of the safety of the nanovesicular systems is promising, because it demonstrates that the mixture of nonionic surfactants in transfersomes does not cause a negative impact on cell viability.

This work is the first indication that the use of optimized proportions of mixed EA in transfersomes is advantageous to refine their physicochemical properties for skin delivery. However, further studies on the efficiency of these nanosystems to penetrate the skin layers, as well as on their safety profile, are necessary to ascertain their relevance for the management of skin conditions. It is noteworthy that these nanovesicular systems may also be incorporated in other DDSs in the future, from semisolid formulations to microneedles, as previously reported in the literature [47,48]. This additional step may significantly improve the performance of the transfersomes to efficiently deliver the drug to the required skin layers. Additionally, it may contribute to a better therapeutic compliance by improving the sensorial experience of the consumers, taking into account that many current topical formulations are ointments that are oily and tacky. In this sense, it can be foreseen that these types of nanovesicular systems may be useful for the management of various skin disorders, as different types of bioactive compounds may be loaded and the composition of the vesicles can be optimized using a QbD design, as described herein for IBU, to ensure the best physicochemical properties for skin delivery.

## 5. Conclusions

This work describes for the first time a QbD strategy to modulate the physicochemical properties of transfersomes for skin delivery based on the incorporation of mixed EAs with opposing HLB values. This approach was found to be valuable, as the optimized nanosystems displayed suitable Vs, PDI, and ZP, with reasonably good EE and LC considering IBU as the model drug. Interestingly, the incorporation of mixed edge activators was an advantage considering the colloidal stability over time under refrigerated conditions. Furthermore, these nanovesicular systems were found to be cytocompatible using a 3D model of human keratinocytes. Therefore, the use of mixed edge activators in transfersomes seems to be a useful strategy to load active compounds for the management of skin conditions.

Additional studies are needed to further support the clinical use of the developed vesicular nanosystems. Some are already ongoing, by probing the in vitro release and skin permeation of both hydrophilic and lipophilic model drugs. Other studies on in vivo safety and efficacy are also foreseen.

## Figures and Tables

**Table 1:** Factors and responses selected for the BBD and their respective tested levels and desirability criteria.

Factors	Levels		
	−1	0	1
X_1_: Lipid concentration (% *w*/*v*)	4	6	8
X_2_: Tween^®^ 80/Span^®^ 80 ratio	2.5:12.5	7.5:7.5	12.5:2.5
X_3_: Ibuprofen concentration (% *w*/*v*)	0.05	0.10	0.15
**Responses**	**Desirability**		
	**Low**	**Medium**	**High**
Y_1_: Vesicle size (nm)	180	130	80
Y_2_: Polydispersity index	0.30	0.22	0.15
Y_3_: Encapsulation efficiency (%)	10	25	40
Y_4_: Loading capacity (%)	0.10	0.45	0.90

**Table 2:** Composition of the prepared formulations and obtained responses (*n* = 3, mean ± SD) for optimizing IBU-loaded transfersomes using BBD.

Formulation #	Factors			Responses			
	X_1_	X_2_	X_3_	Y_1_	Y_2_	Y_3_	Y_4_
1	4	(2.5:12.5)	0.10	172 ± 36	0.17 ± 0.06	35 ± 1	0.88 ± 0.03
2	8	(2.5:12.5)	0.10	155 ± 1	0.26 ± 0.01	38 ± 2	0.50 ± 0.03
3	4	(12.5:2.5)	0.10	85 ± 1	0.27 ± 0.01	24 ± 2	0.63 ± 0.06
4	8	(12.5:2.5)	0.10	126 ± 5	0.28 ± 0.02	26 ± 3	0.34 ± 0.03
5	4	(7.5:7.5)	0.05	136 ± 7	0.21 ± 0.05	20 ± 6	0.26 ± 0.08
6	8	(7.5:7.5)	0.05	148 ± 1	0.18 ± 0.01	23 ± 2	0.15 ± 0.01
7	4	(7.5:7.5)	0.15	103 ± 3	0.26 ± 0.00	19 ± 1	0.71 ± 0.05
8	8	(7.5:7.5)	0.15	132 ± 3	0.19 ± 0.03	16 ± 2	0.29 ± 0.03
9	6	(2.5:12.5)	0.05	161 ± 6	0.23 ± 0.02	34 ± 2	0.34 ± 0.02
10	6	(12.5:2.5)	0.05	98 ± 2	0.28 ± 0.01	10 ± 3	0.08 ± 0.03
11	6	(2.5:12.5)	0.15	136 ± 2	0.26 ± 0.02	27 ± 2	0.67 ± 0.04
12	6	(12.5:2.5)	0.15	167 ± 6	0.29 ± 0.02	16 ± 1	0.32 ± 0.09
13	6	(7.5:7.5)	0.10	127 ± 3	0.19 ± 0.02	16 ± 2	0.26 ± 0.04
14	6	(7.5:7.5)	0.10	131 ± 3	0.18 ± 0.02	12 ± 1	0.19 ± 0.01
15	6	(7.5:7.5)	0.10	121 ± 1	0.19 ± 0.01	18 ± 1	0.30 ± 0.02

X_1_: lipid concentration (% *w*/*v*), X_2_: Tween^®^ 80/Span^®^ 80 ratio, X_3_: IBU concentration (% *w*/*v*), Y_1_: vesicle size (nm), Y_2_: polydispersity index, Y_3_: encapsulation efficiency (%), Y_3_: loading capacity (%).

**Table 3:** Interaction coefficients (Coef.) and respective *p*-values obtained from the regression analyses performed using the two-way interaction (linear × quadratic) model to evaluate the impact of factors and their interactions on the observed responses. The coefficient of determination (R^2^) of each regression analysis is also presented.

	Vs		PDI		EE		LC	
	Coef.	*p*-Value	Coef.	*p*-Value	Coef.	*p*-Value	Coef.	*p*-Value
**Int.**	**134.92**	**0.0001**	**0.240**	**0.0001**	**24.13**	**0.0015**	**0.431**	**0.0014**
X_1_	7.42	0.0584	0.008	0.0607	0.83	0.5692	**−0.156**	**0.0173**
X_1_^2^	0.65	0.6708	−0.001	0.6349	−3.48	0.0551	**−0.084**	**0.0282**
X_2_	**−22.00**	**0.0072**	**0.027**	**0.0064**	**−6.94**	**0.0296**	**−0.119**	**0.0290**
X_2_^2^	−4.73	0.0689	**−0.028**	**0.0028**	**−4.36**	**0.0363**	**−0.084**	**0.0282**
X_3_	−4.50	0.1385	**0.013**	**0.0251**	−1.54	0.3343	**0.146**	**0.0197**
X_3_^2^	−2.35	0.2141	**−0.011**	**0.0187**	1.22	0.2893	0.033	0.1495
X_1_X_2_	**14.50**	**0.0288**	**−0.020**	**0.0202**	−0.28	0.8822	0.023	0.5038
X_1_X_2_^2^	2.13	0.3548	**−0.025**	**0.0066**	−0.68	0.6193	0.018	0.4678
X_1_^2^X_2_	**10.50**	**0.0275**	−0.005	0.1340	−1.60	0.3015	−0.025	0.3318
X_1_X_3_	4.35	0.2333	−0.010	0.0742	−1.33	0.5038	−0.078	0.1084
X_1_^2^X_3_	**11.63**	**0.0226**	−0.002	0.3453	0.88	0.5291	−0.003	0.9106
X_2_X_3_	23.50	0.0113	−0.005	0.2254	3.38	0.1757	−0.023	0.5038
R^2^	0.9943		0.9975		0.9794		0.9917	

X_1_: lipid concentration, X_2_: Tween^®^ 80/Span^®^ 80 ratio, X_3_: IBU concentration, Vs: vesicle size, PDI: polydispersity index, EE: encapsulation efficiency, LC: loading capacity. Statistically significant effects are highlighted with bold style.

**Table 4:** Factors and theoretical responses predicted for the optimized transfersomes, including the responses obtained in the experimental validation.

Optimized Formulation	Responses	Theoretical Responses ^1^	Experimental Responses ^2^
4:(2.5:12.5):0.125 X_1_:(X_2_):X_3_	Vs (nm)	153.1 (130.0–176.3)	166 ± 20
	PDI	0.19 (0.16–0.21)	0.23 ± 0.03
	EE (%)	32.6 (17.5–47.6)	34 ± 9
	LC (%)	0.98 (0.73–1.24)	1.1 ± 0.3

^1^ 95% confidence intervals are shown in parentheses. ^2^ Data are presented as mean ± SD, *n* = 3.

**Table 5:** Physicochemical properties of unloaded and IBU-loaded transfersomes (Unl-TR and IBU-TR, respectively) and liposomes (Unl-Lip and IBU-Lip, respectively) immediately after preparation.

Formulation	IBU (% *w*/*v*)	Tween^®^ 80/Span^®^ 80 ratio	Vs (nm)	PDI	ZP (mV)	EE (%)	LC (%)
[Unl-TR]	0	2.5:12.5	151 ± 17 *	0.25 ± 0.02	−50 ± 3	-	-
[IBU-TR]	0.125	2.5:12.5	166 ± 20 **	0.23 ± 0.03	−47 ± 6	34 ± 4	1.1 ± 0.3
[Unl-Lip]	0	0	106 ± 5	0.27 ± 0.01	−44 ± 2	-	-
[IBU-Lip]	0.125	0	137 ± 20	0.29 ± 0.04	−37 ± 8	30 ± 4	0.9 ± 0.1

Vs, vesicle size; PDI, polydispersity index; ZP, zeta potential; AE, association efficiency; LC, loading capacity. *n* = 3, mean ± SD. * *p* < 0.05 when compared with Unl-Lip; ** *p* < 0.01 when compared with Unl-Lip.

## Data Availability

The data presented in this study are available in this manuscript.
